# Pain in the hours following surgical and rubber ring castration in dairy calves: Evidence from conditioned place avoidance

**DOI:** 10.3168/jdsc.2022-0241

**Published:** 2022-09-30

**Authors:** Thomas Ede, Emeline Nogues, Marina A.G. von Keyserlingk, Daniel M. Weary

**Affiliations:** 1 Animal Welfare Program, Faculty of Land and Food Systems, University of British Columbia, Vancouver, Canada V6T 1Z4

## Abstract

Male cattle reared for beef are typically castrated. Male calves born on dairy farms are increasingly reared for beef, so a better understanding of how to humanely perform this procedure in dairy systems is now required. We studied the short-term affective responses of dairy calves to castration using a conditioned place aversion paradigm. Young Holstein bulls (16 d old, n = 30) were castrated by either rubber ring (n = 15) or surgery (n = 15), and then kept in a visually distinctive recovery pen for 6 h after the procedure. Calves acted as their own control and were sham castrated and allowed to recover for 6 h in another visually distinctive pen. During both castration and sham procedures, calves received a sedative (xylazine, 0.2 mg/kg), local anesthetic (lidocaine, 5 mL), and a nonsteroidal anti-inflammatory (meloxicam, 0.5 mg/kg). After exposure to both treatments (castration and sham), calves were tested for place aversion by providing free access to the 2 pens where they experienced the different treatments. Calves were tested for aversion 48, 72, and 96 h after their last treatment. We recorded how much time they spent in each pen and where they chose to rest. We did not find differences in time spent resting or resting location, suggesting that calves did not form a more negative memory of castration in comparison to the sham procedure. The lack of treatment effects may be due to features of our testing paradigm, including effective multimodal pain control during the 6-h conditioning period and limiting testing to the first days after the procedure.

## Highlights

• Dairy calves did not seem to form a negative memory of surgical or rubber ring castration when provided multimodal pain control.
• We recommend the use of multimodal pain control (sedation, local block, and analgesia).
• Long-term effects of castration were not investigated.

## Graphical Abstract

**Figure:**
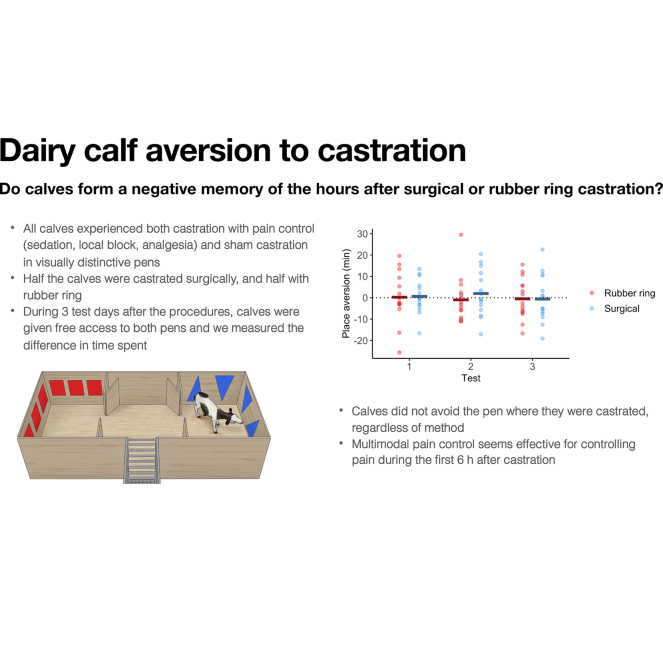
**Summary:** We set out to assess whether calves form a negative memory of two castration procedures (surgical and rubber ring), even when provided with a sedative, local anesthetic, and analgesic. Calves were castrated or shamcastrated in pens with different visual cues (red squares/blue triangles). In the days afterward, we tested if calves avoided the pen where they had experienced the procedure. Surprisingly, calves did not avoid the pen where they had been castrated, suggesting that the multimodal pain control we provided was effective in the hours following castration.

Male calves reared for beef are typically castrated before the age of sexual maturity. Due to several changes in the dairy industry, including the increased use of beef genetics, male calves born on dairy farms are increasingly reared for beef (Poock and Beckett, 2022), but little research has addressed how these calves can be humanely castrated.

Previous research has shown that castration can cause acute and long-term pain (Stafford and Mellor, 2005; Marti et al., 2017; Meléndez et al., 2017b), especially in older animals (Dockweiler et al., 2013). Local anesthesia helps alleviate acute pain (Stewart et al., 2010), and providing a nonsteroidal anti-inflammatory drug (**NSAID**) can mitigate postprocedural pain (Currah et al., 2009; Kleinhenz et al., 2018; Meléndez et al., 2018). Most of the literature on pain due to castration has focused on stress and inflammation biomarkers (Park et al., 2018), weight gains (Bretschneider, 2005), reflex-like behaviors (Meléndez et al., 2018), and activation of the sympathetic nervous system (Stewart et al., 2010). To our knowledge, affective responses (see Ede et al., 2019b) in the days following the procedure have not been studied. We have previously studied the affective pain caused by hot-iron disbudding by measuring how calves remembered the experience, showing that calves avoid the pen where they had been disbudded (Ede et al., 2019a,c). Using a similar paradigm, the main objective of this study was to assess how calves remembered the pain in the days following castration. Our secondary objective was to determine which of 2 common methods of castration (surgery and rubber ring) are remembered as more painful.

This study took place at the University of British Columbia's Dairy Education and Research Centre in Agassiz, Canada, between March and December 2021. The research was approved by the University of British Columbia's Animal Care Committee (application A18–0376).

Based on R's base package “power.t.test” function (R Core Team, 2021), and our previous aversion results presented in a study of disbudding (Ede et al., 2019c), a minimum sample size of 7 calves per treatment group was determined for an anticipated 80% statistical power. Given this was our first study on aversion to castration pain, we opted to increase our sample to 15 animals per treatment.

Thirty Holstein bulls (birthweight: 41.4 ± 5.7 kg) were housed individually in single pens (2.1 × 1.2 m), then paired in double pens (6.1 ± 1.7 d old at pairing). Calves were paired based on similarity in birth date (age difference within pairs averaged 1.5 ± 1.8 d), fed 8 L/d of whole milk across 2 feedings (at approximately 0800 and 1600 h) and provided ad libitum access to hay, grain, and water.

On the first day of trial, calves (16 ± 2.3 d old) were brought individually to the experimental apparatus (Figure 1) for an initial exposure at approximately 1000 h. Calves were given a 0.5 L milk reward in a chute before entering the apparatus. Once inside, they could roam freely for 15 min. One calf did not enter all 3 pens (i.e., with at least both front legs in the pen) during this initial exposure session and was excluded from the study.

**Figure 1:**
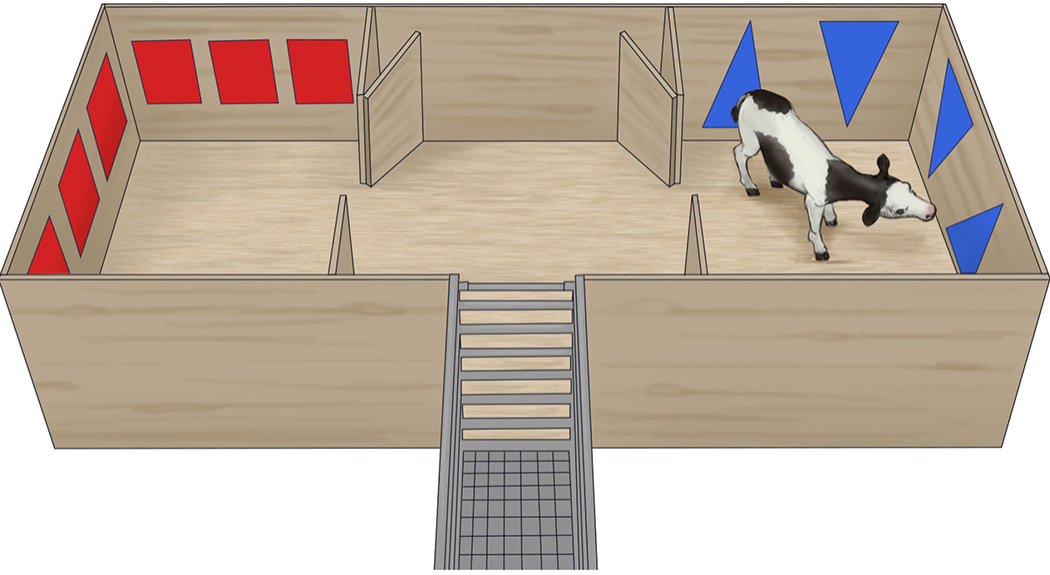
Experimental apparatus (2.1 × 6.0 m). Divided in 3 pens: one neutral pen in the middle and 2 treatment pens on either side (one with red squares and the other with blue triangles on the walls). During initial exposure and aversion tests, calves had access to all 3 pens. During the conditioning treatments (either a castration or sham procedure), calves were restricted to a treatment pen for 6 h. Illustration by Ann Sanderson (independent illustrator, Canada).

Calves received 2 treatments: one 24 h and one 72 h after the initial exposure session (i.e., allowing 48 h to recover between conditioning sessions). All calves experienced both treatments, each in a visually distinctive treatment pen (i.e., if a calf was castrated in the red squares pen, the calf received the sham treatment in the pen with blue triangles, and vice versa). Castration method (surgical or rubber ring), treatment order (castration or sham first), color of castration pen (red squares or blue triangles), and assignment of the preferred pen in the initial exposure session (to castration or sham) were balanced by block and assigned pseudorandomly within block. During each treatment, calves were individually brought to the chute in front of the apparatus, given a 0.5 L milk reward, and injected with a sedative (xylazine, 0.2 mg/kg subcutaneous in rump, Rompun, Bayer). Calves were then led to their assigned treatment pen. Once fully sedated (i.e., recumbent with eye rotation, approximately 10 min after injection), local anesthetic (lidocaine 2%, epinephrine 1:100,000, Lido-2, Rafter8; 1.5 mL in each testicle and 2 mL SC in the scrotal neck) and nonsteroidal anti-inflammatory (meloxicam 0.5 mg/kg, SC in the neck; Metacam 20 mg/mL, Boehringer Ingelheim) were provided. Timing of administration was based on Meléndez et al. (2017a).

For surgical castration, the bottom third of the scrotum was removed, testes were pushed out one at a time, and detached by pulling the base of the spermatic cord. The elongated spermatic cords were cut if protruding, and the wound was left open to drain and heal. For rubber ring castration, an elastrator (Odontomed 2011) was used to place a tight rubber ring around the base of the scrotum, making sure both testes were below the ring. For sham castration, gentle pressure was applied by hand around the scrotal area. Sedation and analgesia were provided, but not local anesthesia (as half the calves had already been castrated).

Two days after the second conditioning treatment calves were tested for place aversion (at approximately 1000 h). Tests were similar to initial exposure session: calves were brought to the apparatus and provided access to all pens. Test sessions ended after 60 min, or when the calf lay down for at least 1 min (whichever came first). Calves were tested 3 d in a row, 48 h, 72 h, and 96 h after their second conditioning session. Observers were not blinded to treatment during aversion tests.

Differences in time spent in the castration versus sham pen during the place aversion test sessions were analyzed with linear mixed models using R lme4 and lmerTest packages (Bates et al., 2015; Kuznetsova et al., 2017; R Core Team, 2021). Fixed effects were castration method (rubber ring or surgical), place aversion test session (1, 2, and 3; continuous), castration pen (red square or blue triangles), and treatment order (castration or sham first). Calf ID was included as a random effect to avoid pseudoreplication. Normality and homoscedasticity of residuals were confirmed graphically. The effect of conditioning treatment on the pen that calves chose to lie down in was analyzed using R base package chi-squared tests (R Core Team, 2021).

As expected, calves showed no preference for one treatment pen over another [95% CI = (−1.6 min, 2.0 min), *P* = 0.8] before conditioning. However, after the conditioning sessions, we still found no evidence that calves avoided the pen where they were castrated [95% CI = (−5.6 min, 7.8 min), *P* = 0.8], regardless of castration method [95% CI = (−4.7 min, 7.1 min), *P* = 0.7; see Figure 2 for details]. We also found no effect of test session [95% CI = (−2.3 min, 1.3 min), *P* = 0.6], color of treatment pen [95% CI = (−7.8 min, 4.0 min), *P* = 0.6] or treatment order [95% CI = (−5.2 min, 6.7 min), *P* = 0.8].

**Figure 2:**
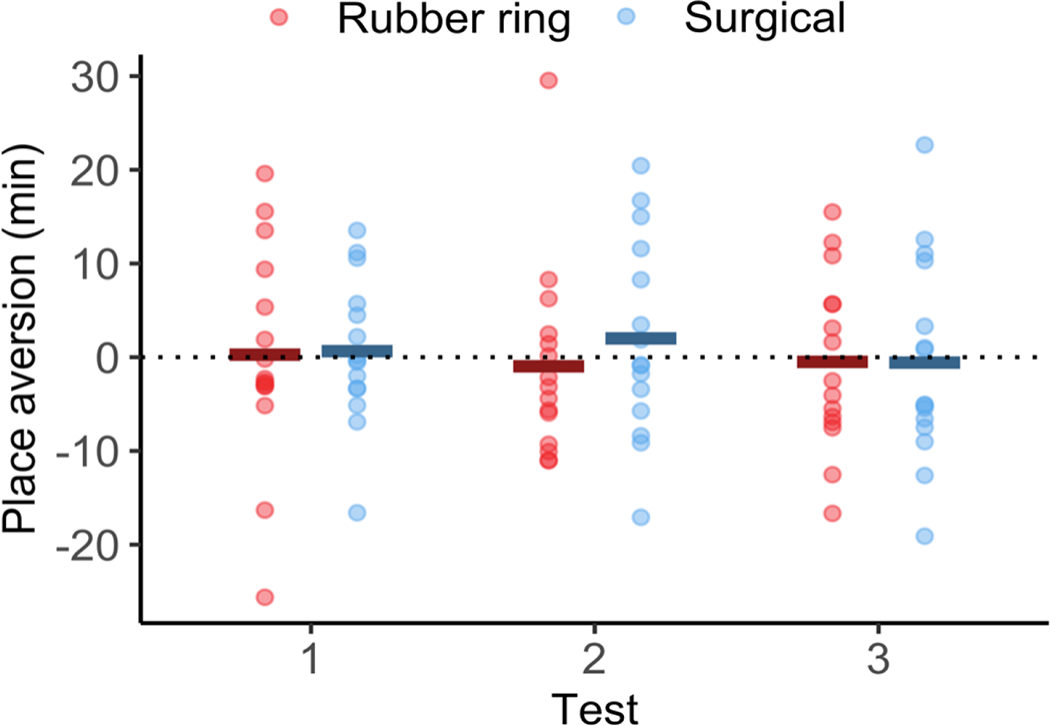
Difference in time (min) spent in the pen where calves were previously castrated and where they received a sham procedure; negative values indicate aversion to the castration pen. Tests 1, 2, and 3 took place 48, 72, and 96 h, respectively, after the calf's last procedure. Calves were treated with a sedative (xylazine), a local anesthetic (lidocaine), and an analgesic (meloxicam) for castration procedure. Dots show values from individual calves, and solid bars show treatment means.

On one occasion a calf did not lie down during the test session (surgical group, first aversion test), but among the remaining tests, we found no evidence that calves avoided lying down in the pen where they had been castrated in comparison to the sham pen (Figures 3A and 3B), regardless of test session or castration method (rubber ring, test 1: χ^2^ = 0.3, *P* = 0.6; test 2: χ^2^ = 0.4, *P* = 0.5; test 3: χ^2^ = 0.1, *P* = 0.7; surgical, test 1: χ^2^ = 1, *P* = 0.3; test 2: χ^2^ = 0, *P* = 1; test 3: χ^2^ = 0.8, *P* = 0.4).

**Figure 3:**
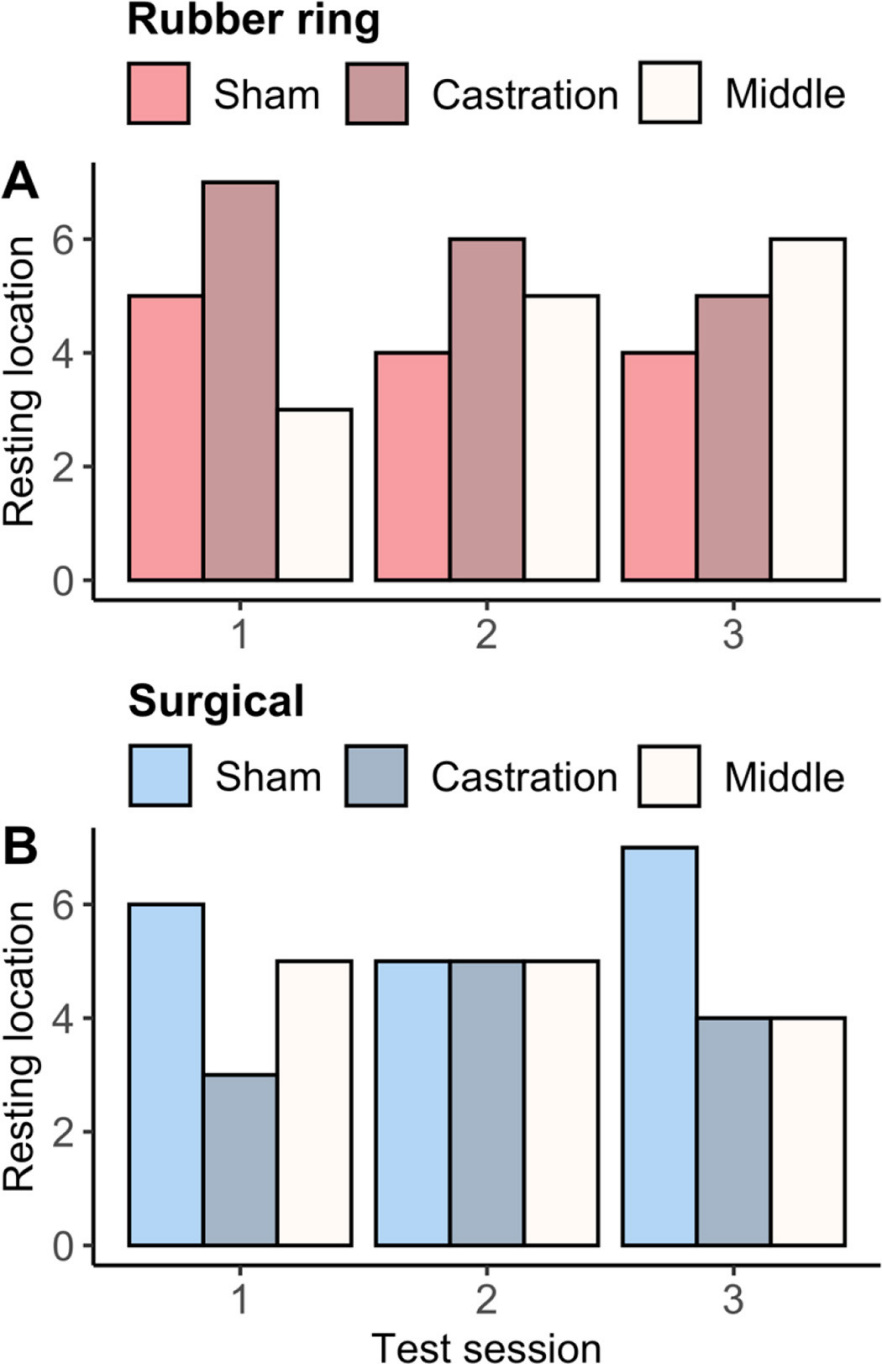
Treatment received in the pen calves chose to lay down during aversion tests (A: rubber ring castration, B: surgical castration). Tests 1, 2, and 3 took place 48, 72, and 96 h, respectively, after the calf's last procedure. Calves were treated with a sedative (xylazine), a local anesthetic (lidocaine), and an analgesic (meloxicam) for castration procedures.

We did not find evidence of conditioned place avoidance (**CPA**) of the pen where calves had experienced castration, regardless of castration method. We had expected calves would experience pain during castration, and the association between this pain experience and the pen features would result in avoidance when tested in the days after the procedure.

Castration is painful (Stafford and Mellor, 2005) as demonstrated by a wide variety of evidence. For example, Molony et al. (1995) found a surge in cortisol in the minutes following both rubber ring and surgical castration, and others (Stewart et al., 2010; Dockweiler et al., 2013) have reported increased eye temperature and electroencephalographic stress response 20 min after castration compared with control calves. Three hours after the procedure, rubber ring and surgical castration induced abnormal postures in calves (Robertson et al., 1994); even with the provision of local anesthesia and the NSAID flunixin, surgically castrated calves display more scrotal licks and tail flicks in the hours after the procedure (Webster et al., 2013). In addition, 2 to 4 h after band or surgical castration, differences were reported in a subjective pain score, leg movements, vocalizations, tail flicks, and suckling behavior (Meléndez et al., 2018). Surgically castrated calves provided with local anesthesia and flunixin have higher cortisol and substance P levels than uncastrated calves in the 6 h after the procedure (Park et al., 2018).

However, some studies have failed to detect differences between castrated and control calves. For example, one study found no differences for 20 min after castration in heart rate variability, eye temperature, cortisol levels, electrodermal activity, and the inflammation marker substance P (Dockweiler et al., 2013). Three hours after the procedure, cortisol in rubber ring and surgically castrated calves were back to levels similar to those of calves who had only been handled (Robertson et al., 1994; Molony et al., 1995). In addition, 2 to 4 h after castration, band or surgical castration did not appear to affect foot stomps, stride length, walking, standing, lying, or eating behaviors (Meléndez et al., 2018).

In the current study, we provided multimodal pain control for ethical reasons, but this likely reduced differences between sham and castration treatments. One previous study that also provided local anesthesia and flunixin to surgically castrated calves found no differences between the castrated and sham treatments 3 h and 8 h following the procedure in cortisol, feeding behavior, foot stomps, grooming, leg lifts, and abnormal lying and standing (Webster et al., 2013). Another study reported that a combination of local anesthesia and the NSAID ketoprofen virtually eliminated the cortisol response to castration (Stafford et al., 2002). Meloxicam is also known to reduce physiological and behavioral responses following castration (Olson et al., 2016). To our knowledge, the current study is the first to assess the short-term effects of castration in calves that had been treated with sedation, local anesthesia, and postoperative analgesia. It seems likely that this combination mitigated pain during the conditioning period, although we suggest it is unlikely that this multimodal treatment was so effective as to make castration indistinguishable from the sham procedure.

It is possible that both castration and sham procedures were aversive to calves. A limitation of the CPA methodology is that it does not indicate if either treatment is positive or negative, only the relative preference between the 2 options. The current results could be due to both treatment pens being associated with a negative experience. Another possibility is that the xylazine sedative used in this study affected memory. In rodents, xylazine has been reported to induce memory impairment in an avoidance task (Morgan and Riccio, 1992). That said, we have previously found that xylazine-treated calves were able to distinguish between painful and sham procedures, with calves avoiding a pen where they had experienced hot-iron disbudding relative to a pen where they had experienced a sham procedure (Ede et al., 2019c). Possibly the contrast between castration and sham castration was less pronounced than that between disbudding and sham disbudding. The study on disbudding was also conducted on older heifers, so differences between the studies could also have been due to age and sex differences as others have found (Bretschneider, 2005; Dockweiler et al., 2013; Marti et al., 2017; Martin et al., 2022; Mogil, 2020). Additionally, castration has been reported to affect calf walking behavior and stride length (Currah et al., 2009; Meléndez et al., 2017b), which could have affected calf willingness to move between the pens and thus interfere with any effects of aversion.

To reduce the number of animals used, we opted for a within-subject design such that each calf acted as its own control and received both castration and sham procedures. Calves who were castrated first were likely experiencing pain during their sham treatment due to the ongoing pain in the days following the procedure (Meléndez et al., 2017b). Palpation following castration has been reported to induce pain in calves following both rubber ring (Becker et al., 2012) and surgical (Norring et al., 2017) castration. We did not observe an effect of treatment order on aversion, perhaps because our paradigm was not sensitive enough to detect this effect.

The 6-h conditioning period only assessed acute effects, but castration is known to have longer term effects. Regardless of method, castration negatively affected weight gain for several weeks (Fisher et al., 2001; Bretschneider, 2005). Meléndez et al. (2017b) noted that surgically castrated 1-week-old calves were affected the day of castration, whereas band-castrated calves started to show changes in lying and standing behaviors only 2 and 3 d after the procedure. Although surgery caused a higher cortisol peak in the minutes after castration, calves castrated with rubber rings had higher lesions scores and lesions licks for 48 d after castration (Molony et al., 1995). Marti et al. (2017) reported a lower weight gain over 68 d for surgically castrated calves compared with control and band calves, despite more intense and longer swelling for band castration calves. In a study with methods most comparable to ours (use of sedative, local anesthesia, and analgesia in Holstein calves <1 mo old), calves castrated with rubber rings took longer to heal, had more inflammation, gained less weight, spent less time lying, and exhibited more lesion lickings than surgically castrated calves (Nogues et al., 2021).

In conclusion, we found no evidence of conditioned place aversion in the 4 d following rubber ring and surgical castration with multimodal pain control consisting of sedation, local anesthesia, and treatment with a postoperative anti-inflammatory.
